# Re-evaluating the placebo response in recent canine dietary epilepsy trials

**DOI:** 10.1186/s12917-024-04066-z

**Published:** 2024-05-24

**Authors:** Teresa Schmidt, Nina Meyerhoff, Sebastian Meller, Friederike Twele, Marios Charalambous, Benjamin A. Berk, Tsz H. Law, Rowena M. A. Packer, Brian Zanghi, Yuanlong Pan, Andrea Fischer, Holger A. Volk

**Affiliations:** 1 Department of Small Animal Medicine and Surgery, University of Veterinary Medicine Hannover, Hannover, Germany; 2 Centre for Systems Neuroscience, University of Veterinary Medicine Hannover, Hannover, Germany; 3 BrainCheck.Pet® - Tierärztliche Praxis für Epilepsie, Mannheim, Germany; 4 Department of Clinical Science and Services, Royal Veterinary College, Hatfield, UK; 5 Research and Development, Nestlé Purina PetCare, St. Louis, MO USA; 6 Centre for Clinical Veterinary Medicine, Ludwig-Maximilians-Universität München, Munich, Germany

**Keywords:** Placebo response, Placebo effect, Honeymoon effect, Crossover trials, Epilepsy, Dogs

## Abstract

The placebo response is a common phenomenon. Limited evidence is available about its magnitude in canine epilepsy trials, even though it can significantly influence the efficacy evaluation of new treatments. It was hypothesised that the placebo response is diminished when epilepsy trials are conducted in a prospective crossover design. Seizure data spanning six months from three previous multicenter epilepsy studies were analysed. The monthly seizure frequency of 60 dogs diagnosed with idiopathic epilepsy was calculated, comparing baseline data with placebo treatment. Furthermore, differentiation was made between dogs randomised to the placebo group early (Phase 1: first 3 months) or later during the study (Phase 2: second 3 months).

The analysis did not reveal any placebo response in terms of monthly seizure frequency. Instead, an increase was noted during the placebo treatment period, with a mean of 2.95 seizures per month compared to 2.30 seizures per month before study entry (*p* = 0.0378). Additionally, a notable phase effect was observed. Dogs receiving the placebo in the second study phase exhibited a significant increase in monthly seizure frequency compared to baseline (*p* = 0.0036). Conversely, no significant difference from baseline was observed for dogs receiving the placebo in the first study phase. These findings underscore the considerable variability in placebo responses observed in trials for canine epilepsy, contrasting with previous limited data. The identified phase effect should be carefully considered in the design and evaluation of canine epilepsy trials to ensure a more accurate assessment of efficacy for new treatments.

**Supplementary Information:**

The online version contains supplementary material available at 10.1186/s12917-024-04066-z.

## Introduction

The placebo response is a commonly observed phenomenon in human and veterinary medicine [1–3]. It is defined as a beneficial health response to administered substances without medical efficacy [1]. In this study, the term ‘placebo response’ refers to an improvement initiated by the administered compound and is also applied as a general term to describe improvements observed in the placebo group, independent of the underlying cause. The mechanism inducing the placebo response has not been conclusively elucidated. A multitude of psychological and neurobiological factors are considered to elicit the phenomenon, such as expectations, conditioning, character traits, neurotransmitters (dopamine, oxytocin, serotonin, norepinephrine, endogenous opioid neuropeptides), hormones (peptide cholecystokinin), immune response and genetics [1, 4, 5].

In animals, the placebo response is assumed to be based on similar mechanisms as in humans, such as conditioning, thereby induced expectancy, and ancillary mechanisms caused by human contact and care [2]. In veterinary medicine, the placebo response has been reported in different species and conditions [6–9]. Overall, these studies have shown that treatment success is evaluated using investigator-defined outcome measures, which induces an inevitable bias. Assessments of clinical improvement in the placebo groups differed between owners and veterinarians [7, 8]. Subjective rather than objective measures are often used. Former studies revealed different outcomes when objective parameters were evaluated [7–9]. In summary, these aspects result in different magnitudes of the placebo response depending on the evaluation method and examiner.

In canine epilepsy, the placebo response has been previously described using data from three clinical trials [3]. Seizure reduction compared to baseline was assessed in the placebo group of a surgical intervention study (crossover design), a novel drug trial (crossover design) and a dietary modification study (parallel design). An overall seizure frequency decrease of 79% relative to baseline occurred in the placebo groups, whereof 29% showed a seizure reduction of more than 50% and were therefore stated as treatment responders [3]. No further analysis was done to elucidate reasons for positive responses in the placebo groups.

The placebo response can have a significant impact on the assessment of treatment efficacy, as it not only affects the clinical reaction to inactive treatments [5, 10]. The effect also extends to active treatments and can disguise their real efficacy [5, 10]. Therefore, the gold standard of clinical trials is currently a placebo-controlled double-blinded study design to assess the effectiveness of novel treatments [1]. In addition, the use of standardised objective outcome parameters should be considered whenever possible. It is crucial to reduce bias and understand its impact in trials to verify the superiority of the intervention over the inert treatment [1]. Thus, three placebo-controlled, double-blinded, crossover canine epilepsy trials were conducted following the gold standard of study design [11, 12]. Unexpectedly, no strong responses in the placebo groups were perceived, and it was hypothesised that the placebo response was diminished in canine epilepsy trials conducted in a prospective crossover design. It is important to elucidate the magnitude of the placebo response in different trial designs to enable an accurate efficacy assessment of the tested treatment and to improve trial designs in the future.

## Materials and methods

Data from three former epilepsy trials at international study sites were assessed in this multicenter study [11–13]. One of these studies is yet to be published, but the placebo data are reported here [13]. The clinical trials evaluated the efficacy of dietary modifications on the seizure semiology of canine idiopathic epilepsy in privately owned dogs. The trials were approved by the local Ethics and Welfare Group (EWG), the Clinical Research Ethical Review Board (CRERB) and the Lower Saxony State Office for Consumer Protection and Food Safety (Niedersächsisches Landesamt für Verbraucherschutz und Lebensmittelsicherheit [LAVES], Oldenburg, Germany) (URN 2011 1132, URN 2016 1558, approval number 33.8-42502-05-19A469). The more recent studies followed the ‘Animal Research: Reporting of In Vivo Experiments’ (ARRIVE) guidelines, where applicable [11, 13, 14]. All trials were designed as prospective, randomised, double-blinded, placebo-controlled crossover studies, therefore offering a comparable placebo arm with the same patients. Identical inclusion criteria for the patients were applied at all sites. All dogs had an unremarkable magnetic resonance imaging of the brain and met the requirements of Tier II confidence level of the International Veterinary Epilepsy Task Force (IVETF) for the diagnosis of idiopathic epilepsy [15]. Two modifications to the IVETF criterion were applied: the maximum age at seizure onset was increased to 12 years and abnormalities in the interictal physical and neurological examination caused by antiseizure medication (ASM) were tolerated. Patients received at least one long-term ASM in a steady state and had experienced at least three generalised epileptic seizures under their current medication in the past three months prior to study participation. The first study tested a medium chain triglyceride (MCT) - enriched diet, the second one a MCT dietary supplement, whereas the latest unpublished study assessed a probiotic supplement as dietary intervention [11–13]. Each dog received the randomised allocated diet or / dietary supplement (intervention vs. matching placebo) for three months alongside their current therapeutic treatment, followed by a switch to the respective other diet or / dietary supplement (crossover) administered for another three months. No changes of the current long-term ASM were allowed, but voluntary withdraw from the study was possible the entire time [11, 12]. In the unpublished study, a dose adjustment of the current ASM after serum concentration assessment was allowed [13]. The introduction of a new long-term addon ASM resulted in exclusion in all the three studies [11–13]. Between crossovers, a wash out period of one week for the MCT dietary supplement and of three weeks for the probiotic supplement was applied [11, 13]. No wash out period was applied in the first MCT - enriched diet study [12].

Evaluation of the seizure semiology was based on daily owner reports, documented in standardised seizure diaries. Furthermore, individual seizure reports logged by owners for three months prior to study participation were assessed as baseline data. The seizure diaries and reports were used to determine the seizure frequency and differentiate the seizure types, according to the definition of the IVETF consensus report (focal epileptic seizures, generalised epileptic seizures, cluster seizures, status epilepticus) [16]. For statistical analysis, only convulsive seizures were considered. Estimating the seizure frequency, the number of seizures per month was calculated for the baseline period prior to study entry and for the placebo phase during the trial, with an additional distinction between the placebo administration in the first and second study phases. In the case of cluster seizures (> 1 seizure within 24 h), single seizures were counted.

Statistical analyses were conducted utilising GraphPad Prism 9 (GraphPad Software, Inc., La Jolla, CA, USA) to test the hypothesis that there is a diminished placebo effect in canine epilepsy crossover trials. Data were tested against the hypothesis of normal distribution using the Shapiro-Wilk test. Data were mainly not normally distributed. Group comparisons were analysed with a Wilcoxon’s signed-rank test for paired data or a Mann Whitney U-test for unpaired data. All tests were two-sided and a *p* ≤ 0.05 was considered statistically significant. Parametric data are presented as mean and standard deviation (SD), and non-parametric data as median and range.

## Results

### Study population

Sixty dogs were included in this study, which were former participants of three previous dietary epilepsy trials, two published [11, 12] and one just finalised. The study population consisted of 37 males (neutered, *n* = 25; intact, *n* = 12) and 23 females (neutered, *n* = 15; intact, *n* = 8). The mean age was 4.8 (SD 2.2) years and the mean weight was 28.43 (SD 15.5) kg at the first visit of the trial. Thirty-five different breeds were included, with Border Collie (*n* = 5) and Beagle (*n* = 3) being the most common ones, as well as 12 cross-breed dogs [see Additional file 1]. All dogs were chronically treated with at least one ASM. Of the 60 dogs, 57 received phenobarbital (95%) and 39 were treated with potassium bromide (65%). Phenobarbital was administered as monotherapy in 19 dogs. Potassium bromide was administered as monotherapy in one dog and as an adjunctive therapy to phenobarbital in 38 dogs. Imepitoin was administered in seven dogs (11%), as monotherapy in two of these dogs. Additional routine ASM treatment was levetiracetam (*n* = 24), retigabine (*n* = 1) and gabapentin (*n* = 1). Rescue therapy was oral levetiracetam pulse (*n* = 12) and rectal diazepam (*n* = 32). Four dogs of the probiotic study received MCT supplementation (diet/oil) at study entry. Overall, polytherapy of routine ASM was administered in 47 of the included dogs (78%), with 25 dogs treated with two routine ASM (42%) and 22 dogs treated with three routine ASM (37%), rescue therapy and dietary supplementation not included [see Additional file 1].

### Placebo response

**Fig. 1:**
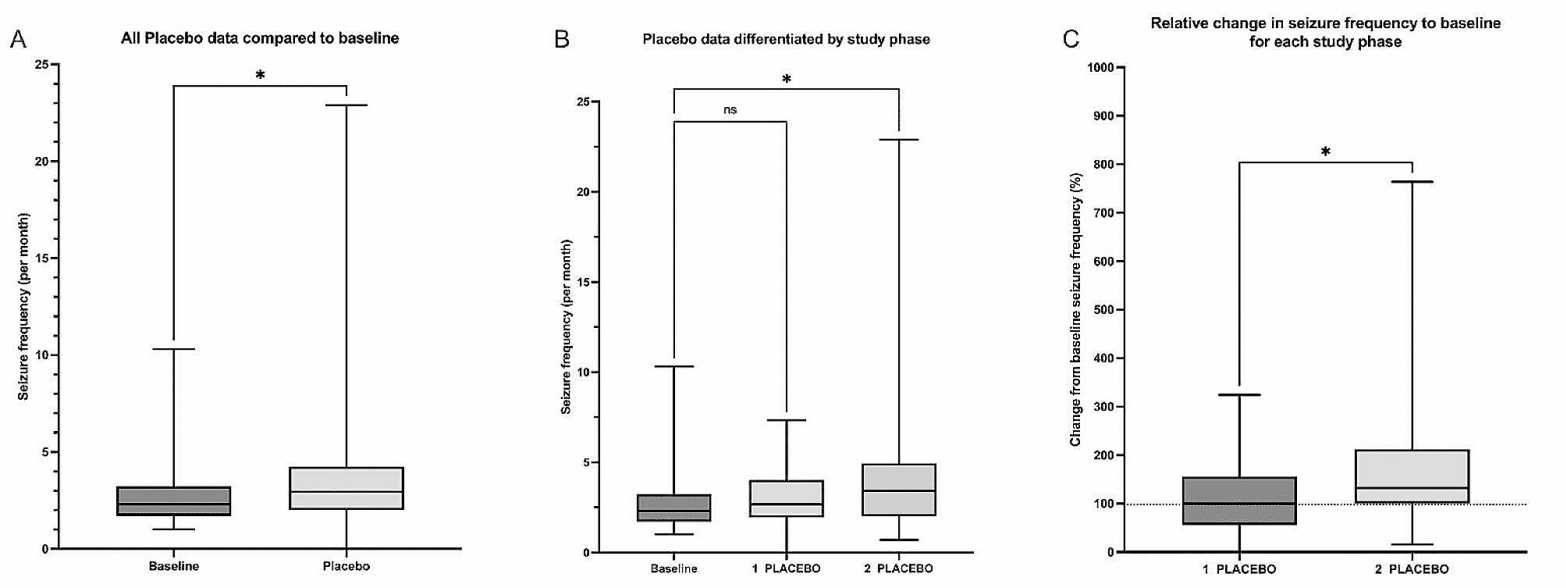
Seizure frequency during baseline and placebo administration of three former dietary canine epilepsy trials. **(A)** No placebo response was found. Monthly seizure frequency of patients increased during participation in the placebo phase compared to baseline (*p* = 0.0378). **(B)** Seizure frequency per month during baseline and the placebo administration differentiated by study phase (1 placebo: 1–3 study months; 2 placebo: 4–6 study months). Dogs receiving the placebo in the second study phase showed a significantly increased seizure frequency compared to baseline (*p* = 0.0036). However, no significant difference to baseline was found in the first study phase **(C)** When comparing the relative change in seizure frequency from the two different placebo phases to baseline (100%), seizure frequency in the second study phase was significantly increased (*p* = 0.0171). Two-sided Wilcoxon’s signed-rank test (paired data) and Mann Whitney U-test (unpaired data) were used for comparison

The overall monthly seizure frequency was significantly increased during placebo administration compared to the seizure baseline (*p* = 0.0378, Fig. 1A). The baseline seizure frequency had a mean of 2.30/month (1–10.3/month) and was increased to 2.95/month (0–22.9/month) during placebo treatment (Fig. 1A) [see Additional file 2]. Due to the crossover design, dogs randomly received the placebo treatment either in the first (1 placebo: 1–3 months) or in the second study phase (2 placebo: 4–6 months) of the trial. Differentiating the placebo treatment by study phase revealed a significant phase effect (Fig. 1B). The monthly seizure frequency significantly increased in the second study phase placebo group compared to the baseline (*p* = 0.0036, Fig. 1B), but not in the first study phase. When comparing the relative change in seizure frequency from the two different placebo phases to the baseline, the second phase had a significant increase compared to the first one (Fig. 1C, *p* = 0.0171), highlighting a potential ‘honeymoon effect’ at the beginning of the trial. Seven of the 33 dogs receiving placebo in the first study phase had more than a 50% reduction in seizure frequency (21% partial responders [*n* = 7/33]), with one becoming seizure free (3% responders [*n* = 1/33]), in contrast to only one dog out of 27 dogs (4% partial responders [*n* = 1/27]) receiving placebo in the second study phase showing more than a 50% reduction (Table 1). Overall, of the 60 dogs in this study 68% (*n* = 41/60) had cluster seizures during the baseline and 76% (*n* = 25/33) in the first study phase and 78% (*n* = 21/27) in the second study phase, respectively.

**Table 1:** Number of dogs with relative changes in seizure frequency during placebo administration in the first or second study phase compared to baseline

	1. Placebo	2. Placebo
Partial responder ^1^	7/33 (21%)	1/27 (4%)
Seizure free	1/33 (3%)	0/27 (0%)

^1^ Partial responders: dogs with a > 50% reduction in seizure frequency

## Discussion

The placebo response is a well-recognised phenomenon in veterinary medicine [2]. It has been described in canine epilepsy, where patients responded to placebo treatments with a decrease in seizure frequency of around 30% [3]. However, in three recent dietary placebo-controlled, double-blinded, crossover canine epilepsy trials, such a strong placebo response was not observed [11–13]. It was hypothesised that the placebo response is diminished in canine epilepsy trials conducted in a prospective crossover design. Here, the placebo response is reported in comparison to the retrospective baseline seizure frequency, as previously published [3]. Statistical analysis revealed no placebo response that improved monthly seizure frequency in the conducted trials. In fact, the monthly seizure frequency of placebo treated patients increased during the second study phase compared to the baseline and to the placebo treated patients in the first study phase. However, it is important to note that 21% of placebo-treated dogs were classified as partial responders in the first study phase (1–3 months), whereas 4% of the placebo-treated dogs were considered partial responders in the second study phase (3–6 months). These findings highlight a potential ‘honeymoon effect’ that is already known from ASM treatment in humans and dogs and which, according to our analysed studies, can also occur with placebo treatment [17, 18].

### Placebo responder rates in canine epilepsy

Data evaluating the placebo response in canine epilepsy remain limited to a few previous investigations [3, 19]. One study assessed data from three former epilepsy trials and found a summarised placebo responder rate of 29%, with responders being defined as > 50% reduction in seizure frequency [3]. Another study evaluated data from a former drug trial and also showed an improvement in seizure frequency in the placebo group, but not significantly compared to baseline [19]. The partial placebo responder rate in the first phase of the current study was lower compared to the previous literature (21% of dogs with a > 50% reduction in seizure frequency) and further decreased when dogs received the placebo in the second study phase (4% of dogs with a > 50% reduction in seizure frequency). In our analysed trials, there was no significant change in seizure frequency compared to baseline in the first study phase and even a 60% increase in seizure frequency in the second study phase. Applied outcome parameters between the different studies vary. Defining responders as more than a 50% reduction in seizure frequency or defining responders as seizure freedom should be considered when interpreting and comparing study results.

### Factors influencing the placebo response magnitude

There was significant heterogeneity in the placebo responder rates in canine epilepsy trials within the previous literature and compared to the current findings [3, 19]. In one of these earlier studies, the rate of positive placebo responses also varied widely within different study designs [3]. In the investigated parallel study, a 60% responder rate and a 46% overall seizure frequency decrease were detected [3]. In the two investigated crossover studies, 0% and 36% responder rates, and 26% and 29% overall seizure frequency decrease were revealed [3]. In contrast, in the current study, a long-term increase in seizure frequency and a decrease in responder rate were observed. The magnitude of the placebo response varied within the study phases of placebo administration (1 phase: 21% partial responders and 3% responders; 2 phase: 4% partial responders).

These discrepancies can be caused by several factors that influence the responses in the placebo group [20]. Although they have only been assessed in humans, it is likely that they also affect the placebo response in canine epilepsy. The first of them is a steady increase in placebo responder rates over the last decades [21, 22]. Individual characteristics of the participants, such as age and geographic region, can also result in altered placebo responder rates between trials [22–25]. Additionally, variations in the study design can have an impact, such as different outcome measures (binary vs. continuous variables), duration of the test period and exclusion of non-responders in result assessment [21, 22, 26, 27]. Solely considering the later maintenance phase by exclusion of the former titration phase in epilepsy medication trials or a general long study duration increased the placebo response in humans [21, 22]. In contrast, the observed phase effect in the current study showed a decrease in the placebo response during the last phase of the study period., (Fig. 1B and C). Finally, heterogenic factors of the disease itself, such as time of disease onset, variations in baseline seizure frequency, amount of prescribed ASM, or modification in diet with an influence on baseline drug serum concentration can modify the placebo responder rates [22, 25, 28, 29]. In human medicine, a long epilepsy history, a high median seizure frequency, as well as ASM polytherapy, are predictors of lower placebo responder rates [22, 25, 28]. In canine epilepsy, cluster seizures are a predictor of a general low treatment responsiveness [30]. These factors can indicate severe epilepsy phenotypes of drug-resistant patients. Those patients are likely to participate in new antiepileptic drug trials but might be prone to have a low placebo response due to many therapeutic failures in the past [31]. Most of the included dogs in this study meet these factors that predict lower placebo responses in humans and dogs, which might have had a role in the missing placebo response.

### ‘Honeymoon’ effect

Differentiating the placebo treatment by study phase showed that a significant increase in seizure frequency occurred in dogs receiving the placebo in the second study phase, compared to baseline and the first placebo administration phase (Fig. 1B and C; Table 1). These findings indicate that the deterioration of epilepsy during the trial correlated with time and was progressive in nature. The lack of a significantly increased seizure frequency in the first placebo administration phase might be caused by a small underlying placebo response functioning as a stabilising factor. This would be in accordance with typical characteristics of placebo responses such as early onset, quick interruption and no persistence over a longer period [32]. As a ‘honeymoon effect’, the small placebo response might have delayed the disease progression in dogs receiving the placebo in the first three months of the trial, whereas the placebo response had already decreased in dogs treated with the placebo in the fourth to sixth months of the trial, resulting in a significant increase in monthly seizures. Discrepancies between the magnitude of the placebo responses in the first and second placebo phases might have arisen from higher owner expectations at trial entry, which faded over time.

Another explanation can be conditioning effects evoked by the crossover design itself [33]. Perceptions of the efficacy of the first phase of treatment can create expectations towards the efficacy of the second treatment which persist beyond the washout [33]. This conditioning may also arise in owners participating in crossover trials with their dogs and might be reflected as a phase effect in the current study.

The phase effect during the placebo treatment should be considered in the evaluation and design of epilepsy trials in the future. Even if it was not observed in active treatment [11, 12], it might bias the outcome of direct comparisons between active and inert treatment and may result in a false evaluation of treatment efficacy. To minimise the initial honeymoon effect of the first three months, an overall trial duration of at least six months or one year should be considered. Studies in a crossover design could provide a great advantage due to their randomised group allocation and comparison between both study arms, potentially neutralising the honeymoon effect. Therefore, in crossover studies, a shorter period of three months per study phase could be sufficient. One disadvantage of crossover designs remains. In the case of early dropouts during the first study phase, an intention to treat analysis is challenging. However, exclusion of the non-responding dropout patients in the statistical analysis can cause a positive bias of the results [27]. Many owners are not willing to wait for the switch-over time point when the condition of their pet is deteriorating. Therefore, depending on the owner, it is recommended to change non-responding patients earlier to the respective other phase, instead of losing patients during the trial. This would be one way to enable intention to treat analysis in the statistical evaluation of crossover designed trials and ensure the reliability of the result.

### Limitations

The main limitation of the current study is the use of retrospective seizure data as a baseline, compared to prospective seizure data. The change in documentation format from individual owner records to standardised seizure diaries during the trial may have influenced the results and a retrospective recall error might have occurred. Comparing prospective seizure data to retrospective baseline data proved to be difficult previously [19]. However, obtaining prospective seizure data for baseline evaluation also remains challenging. High dropout rates due to ethical issues caused by a lack of initial treatment of affected dogs and low owner compliance complicate such a study design.

The assessment of seizure frequency based on owners’ reports is another limitation. Daily seizure record are essential, but seizure diaries are often completed retrospectively prior to study visits, which can cause inaccuracies [34]. Another problem for owners is recognising subtle seizure types such as focal epileptic seizures or seizures in their absence [35]. Therefore, only the more obvious generalised seizures were evaluated in this study, usually even detectable after absence due to urine or saliva residues. In the future, technical devices that automatically detect and record seizures might be a valuable option, but they are not fully developed yet and have a low detection sensitivity in dogs [34, 36, 37]. Considering solely convulsive seizures can reduce the placebo response in humans, which may also be the reason for the missing placebo response in this canine trial [38].

Finally, owner and investigator expectations might have influenced the results of the current study. By conducting all three trials in a double-blinded design, this factor was minimised. However, in an early study on antidepressants in humans, high rates of guessing accuracy for group assignment were reported and the authors recommended recording the respective guesses, which should also be implemented in future canine epilepsy trials [39].

## Conclusion

Placebo responder rates in canine epilepsy trials are highly heterogeneous and a large variance can significantly affect the efficacy evaluation of the tested treatment [3, 19, 40]. Consistent epilepsy trials should be conducted in prospective designs, with extended follow-up periods and standardised definitions of drug-resistance and treatment success to gain reliable data of placebo and treatment responses in dogs, comparable to recent approaches in human medicine [41]. Access to this data could pave the way to create computer trial simulations, already conducted in human epilepsy and other neuropsychiatric disorders [42, 43]. In the future, an extended knowledge of the placebo response can improve the clinical trial design and enhance the detection of effective treatments [20].

### Electronic supplementary material

Below is the link to the electronic supplementary material.

Additional file 1: Table 1 Characteristics of the study population and applied medication

Additional file 2: Table 2 Seizure data of the three evaluated epilepsy trials

## Data Availability

All data generated or analysed during this study are included in this published article and its supplementary information files. Further inquiries can be directed to the corresponding author.

## Declarations

### Conferences

Preliminary results of the current study were presented at the following conferences: 1^st^ Texas A&M Neural-Gut Axis Symposium 2024 (awarded with the 3^rd^ poster price).

## Abbreviations

EWG: Ethics and Welfare Group
CRERB: Clinical Research Ethical Review Board
LAVES: Lower Saxony State Office for Consumer Protection and Food Safety (Niedersächsisches Landesamt für Verbraucherschutz und Lebensmittelsicherheit)
ARRIVE: Animal Research: Reporting of In Vivo Experiments
IVETF: International Veterinary Epilepsy Task Force
ASM: antiseizure medication
MCT: medium chain triglyceride
SD: standard deviation
